# The synthesis of nanofiber membranes from acrylonitrile butadiene styrene (ABS) waste using electrospinning for use as air filtration media†

**DOI:** 10.1039/c9ra04877d

**Published:** 2019-09-30

**Authors:** Akmal Zulfi, Dian Ahmad Hapidin, Muhammad Miftahul Munir, Ferry Iskandar, Khairurrijal Khairurrijal

**Affiliations:** Department of Physics, Faculty of Mathematics and Natural Sciences, Institut Teknologi Bandung Jalan Ganesa 10 Bandung 40132 Indonesia miftah@fi.itb.ac.id krijal@fi.itb.ac.id; Research Center for Biosciences and Biotechnology, Institute for Research and Community Services, Institut Teknologi Bandung Jalan Ganesa 10 Bandung 40132 Indonesia; Research Center for Nanosciences and Nanotechnology, Institute for Research and Community Services, Institut Teknologi Bandung Jalan Ganesa 10 Bandung 40132 Indonesia

## Abstract

Acrylonitrile butadiene styrene (ABS) waste has been successfully recycled into nanofiber membranes by an electrospinning method for air filter applications. The ABS precursor solutions were made by dissolving the ABS waste in three different solvents, DMAc, DMF, and THF, with various concentrations of 10, 20, and 30 wt%. The solvent and solution concentrations affected the fiber properties (size and morphology) and membrane properties (wettability, crystallinity, and mechanical). Accordingly, we tested the fabricated membranes using SEM, FTIR, XRD, water contact angle, and tensile strength test measurements. The SEM images depicted three different morphologies, *i.e.* beads, beaded fibers, and pure fibers. The FTIR spectra showed that the solvents completely evaporated during the electrospinning process. The water contact angle test exhibited the hydrophobic properties of all the membrane samples. The XRD spectra showed the amorphous structures of all the membranes. The tensile strength test showed that the membranes fabricated using DMF and DMAc solvents had the best mechanical properties. Considering the fiber size, wettability, and mechanical properties, the membranes fabricated using DMAc and DMF solvents had the best criteria as air filter media. Filtration tests on the membranes fabricated using DMAc and DMF solvents with various solution concentrations depicted that the beads affected the membrane pressure drop and efficiency. The beads gave more space among the fibers, which facilitated the air flow through the membrane. The beads greatly reduced the pressure drop without an overly reduced membrane filtration efficiency. This led to a high-quality factor of the membranes that demonstrated their applicability as potential air filter media.

## Abstract 2

Acrylonitrile butadiene styrene (ABS) waste has been successfully recycled into nanofiber membranes by an electrospinning method for air filter applications.

## Graphical abstract

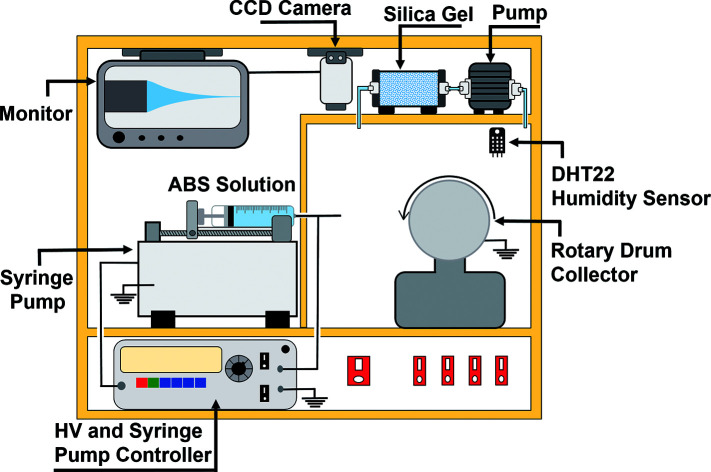

## 1. Introduction

Acrylonitrile butadiene styrene (ABS) is one of the most successful engineered thermoplastic polymers, and is formed from the reaction of three monomers, namely acrylonitrile, butadiene, and styrene.^1,2^ ABS has excellent mechanical properties, dimensional stability, and resistance to chemicals. These properties are utilized in industry, motor vehicles, electronics, and building materials.^1–3^ The ABS is applied in 3D-printer filaments,^4^ computer casings,^2^ printer casings,^5^ and air conditioner housings.^6^ The extensive uses of ABS increase its waste. The worldwide production of the Waste Electrical and Electronic Equipment (WEEE) plastic waste was reported to reach 54 million tons in 2014 and it increased to 70 million tons in 2017 in which the ABS-derived products were the dominant waste.^7–9^ The conventional polymer waste handlings, such as incineration and landfill, create other environmental problems that threaten many organisms.

On the other hand, the recycling process of polymers, especially ABS, can reduce waste and, at the same time, increase their value. Studies related to ABS waste recycling have been carried out by many researchers. Mao *et al.* developed a hybrid material based on the recycled ABS waste,^3^ Sun *et al.* investigated the feasibility of using ABS waste to manufacture reproduction composites.^5^ Palos *et al.* modified cement mortar using recycled ABS.^10^ Recently, some common polymeric waste, such as PET, HIPS, and EPS, were recycled into nanofiber membranes for air filter applications.^11–13^ The nanofiber membranes made from waste materials showed good and promising air filtration performances compared to the membranes made from pure polymers.^11–13^ However, based on our knowledge, the recycling of the ABS waste to give a nanofiber membrane for air filter applications has not been studied in any literature.

Nanofiber-based membranes have small, tortuous, and interconnected pore structures that effectively trap airborne particles while, at the same time, keep the pressure drop low.^14,15^ As an example, Zhang *et al.* reported that polyamide nanofiber membranes exhibited a filtration efficiency of up to 99.97% for PM_2.5_ (particulate matter with an aerodynamic diameter less than 2.5 μm) capture with a pressure drop about 80% lower than those of commercial high efficiency filters.^16^ The low pressure drop membranes are beneficial for many practical applications because they require less energy to pass air through the membranes. Unfortunately, increasing the efficiency of filter membranes is usually followed by an increase in their pressure drop. For example, Zaatari *et al.* reported that replacing a heating ventilation and air-conditioning (HVAC) filter (MERV8) with a higher efficiency filter (MERVE13/14) increased the unit fan power to 11–18% due to the differences in the pressure drop of the filters.^17^ Until now, fabricating air filter membranes that exhibit a high-filtration efficiency with a low-pressure drop is still a challenging issue.

The nanofiber membranes can be fabricated by the electrospinning technique, which utilizes a high electric field to create very long and continuous nanofibers from various polymeric solutions.^18–21^ This technique can control the membrane fiber size and morphology by setting the solution, processing, and environmental parameters to obtain the membranes with the desired fiber properties.

Therefore, this paper comprehensively discusses the ABS waste recycling process using the electrospinning technique. The product of the recycling process is a nanofiber membrane, suitable for air filter application. We studied the effect of the ABS solution concentration and the type of solvents (DMF, DMAc, and THF) on the fabricated nanofiber morphology and diameter. Some solution parameters were measured for all the ABS precursor solutions, including viscosity, surface tension, and conductivity. We also measured the properties of the fabricated nanofiber membranes, including fiber size and morphology, functional groups, crystallinity, wettability, and mechanical properties. The fabricated nanofiber membranes were tested to capture particles in the PM_2.5_ category. We used incense smoke as the test particle.^16^ The results showed that the ABS membrane fabricated using the DMAc solvent with a concentration of 20 wt% had a beaded fiber morphology and the best filtration performance with the lowest pressure drop and highest quality factor value. This result shows the great potential of the ABS waste membranes for their application in masks and air filters.

## 2. Experimental

### 2.1 Materials

The ABS material was collected from the waste of 3D printer filaments (CCTREE, ABS 3D printer filament), the filament was purchased from Shenzhen Primes Technology Co., Ltd, China. We used three solvents to make the precursor solutions, *i.e. N*,*N*-dimethylformamide (DMF), *N*,*N*-dimethylacetamide (DMAc), and tetrahydrofuran (THF). All the solvents were purchased from Sigma Aldrich, Singapore. The selection of solvent is important in the electrospinning process because it directly affects the solution properties (viscosity, conductivity, and surface tension) as well as the fabricated nanofiber membrane properties.

### 2.2 Preparation of precursor solutions for electrospinning

The synthesis of the precursor solutions started with the cleaning of the ABS waste with water and drying at room temperature. Then, the clean and dry ABS waste was cut into pieces, and dissolved in the DMF, DMAc, or THF solvents with concentrations of 10, 20, and 30 wt%. Finally, the solutions were stirred for ∼6 hours at room temperature until they turned into a homogeneous solution.

### 2.3 Characterization of the precursor solutions

The conductivity, surface tension, and viscosity of the precursor solutions were measured at room temperature using a conductometer (Mettler Toledo, Seveneasy Conductivity, Switzerland), du Noüy ring tensiometer (Fisher Scientific, Surface Tensiomat model 21, USA), and Ostwald viscometer (Fisher Scientific, 50 A643, USA), respectively.

### 2.4 Synthesis of nanofibers

**Fig. 1:**
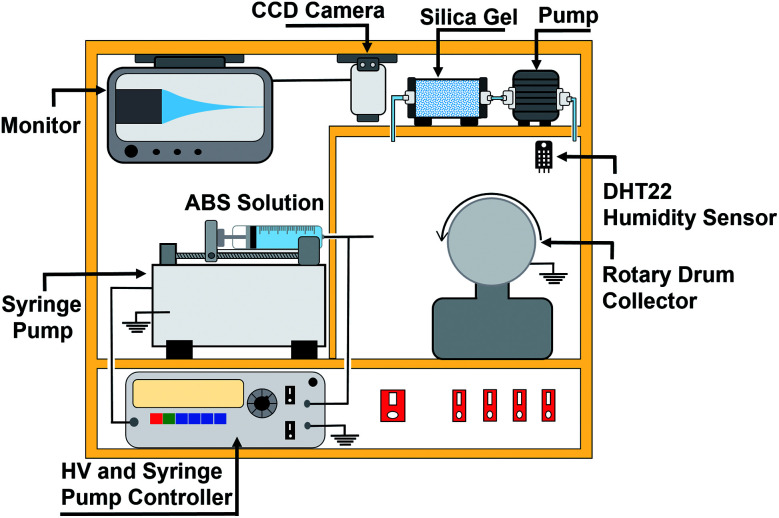
A schematic diagram of the electrospinning setup.

ABS nanofiber membranes were made using an electrospinning apparatus (Nachriebe, Nachriebe 601, Integrated Laboratory of Materials and Instrumentation, Department of Physics, ITB, Indonesia). The electrospinning apparatus, as shown in Fig. 1, consisted of a high voltage source, drum collector, syringe pump, camera and monitor, and a synthesis chamber with a controlled humidity. The precursor solutions were put in a 10 mL syringe with a needle diameter of 0.8 mm. The filled syringe was installed on the syringe pump, which pushed the solution out of the syringe needle with an adjustable flow rate. The needle was connected to a high voltage (HV) source, while the drum collector was grounded. The fabricated nanofibers were deposited on a stainless-steel mesh wrapping the drum collector. A camera with a monitor displayed the Taylor cone formation on the tip of the needle. The processing parameters during the electrospinning process were as follows: high voltage of 15 kV, needle tip to collector distance of 15 cm, solution flow rate of 0.5 mL h^−1^, and humidity of 70%.

### 2.5 Characterization of nanofiber membranes

#### 2.5.1 Fiber/beads size and morphology

The size and morphology of the fabricated fiber/beads were observed using a scanning electron microscope (SEM JEOL-JSM-6510LA, Japan) with 2000- and 4000-times magnification. The fiber size distribution was obtained by randomly measuring the diameter of 100 fibers for each sample. The fiber uniformity was determined from the coefficient of variation (CV) given as follows:

1 CV = *σ*_f_/*μ*_f_

where *σ*_f_ is the standard deviation and *μ*_f_ is the average fiber diameter.^22^ The beads size distribution was obtained by randomly measuring the diameter of 100 beads for each sample. Then, the average bead diameter (*μ*_b_) and standard deviation (*σ*_b_) were taken.

#### 2.5.2 Membrane wettability

The wettability of the electrospun nanofiber membranes was characterized using a contact angle meter (Nachriebe, Nachriebe 320, Integrated Laboratory of Materials and Instrumentation, Department of Physics, ITB, Indonesia) in ambient conditions. The contact angle meter utilized the sessile drop method, where 5 μL of a water droplet was dropped on the membrane surface. Then, the shape of the droplet on the membrane was captured by a camera and the image was digitally processed to determine the water contact angle (CA). The value of the CA was from an average of five repetitive measurements at five different locations above the membrane surface.

#### 2.5.3 Fourier-transform infrared (FTIR) spectroscopy

The FTIR spectra from the cleaned ABS waste and the electrospun nanofiber membranes were obtained using the Fourier-transform infrared (FTIR) spectrometer (Bruker, Alpha 1-176-396, Germany). The FTIR measurements were done in the wavenumber range 600–3600 cm^−1^.

#### 2.5.4 Mechanical property

The mechanical properties of the ABS nanofiber membranes were determined from the stress–strain curve of the tensile strength tests. The tensile strength tests were carried out using a tensile tester (Textechno, Favigraph, Germany). All the tested membranes were previously prepared by cutting into rectangular shapes (25 mm × 2.5 mm) without their substrate. Then, the membrane thickness was measured by a digital micrometer (Sylvac, S228, Switzerland). The membrane thickness must be known to calculate the stress from the tensile strength test data. The extension rate for the testing was 1 mm min^−1^ with a load cell of 100 cN. The tests were done in room conditions (*T* = 25 °C and RH = 70%). The ultimate tensile strength (UTS) was taken as the highest stress during the test. Then, the elongation at break (*dl*) was taken as the highest strain during the test. The Young's modulus was taken as the initial linear slope of the stress–strain curve.

#### 2.5.5 X-ray diffraction

The X-ray diffraction pattern of the cleaned ABS waste and the ABS nanofiber membranes were recorded using an X-ray diffractometer (PANalytical X'Pert Pro, PW3040/X0, Netherlands) with the following measurement conditions: radiation source used Cu target tube; the current was 30 mA; the voltage was 40 kV; the diffraction pattern was recorded at a 2*θ* position in the range of 10 to 45°.

### 2.6 Air filtration performance

**Fig. 2:**
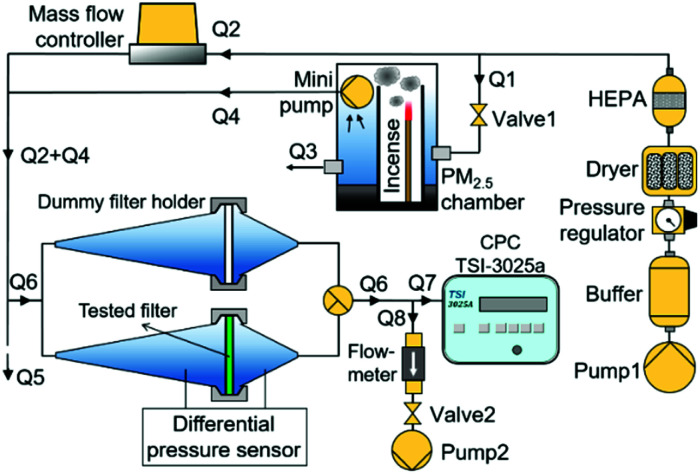
A schematic diagram of the air filter test system.

Fig. 2 depicts the air filter test setup to measure the filter pressure drop, efficiency, and quality factor. The setup consisted of air flow supply and control, filter holder and dummy filter holder, differential pressure sensor, condensation particle counter (CPC), and PM_2.5_ chamber. The PM_2.5_ chamber generated particles in the PM_2.5_ category for filter efficiency measurements. PM_2.5_ is a major concern in many pollution and atmospheric studies due to its penetrability to the respiratory system, which can damage the lungs and cause many health problems.^16,23^ PM_2.5_ generated from the PM_2.5_ chamber originated from the burning incense smoke. The burning incense has been reported to produce smoke in the PM_2.5_ size category and it also represents real pollutants during hazy conditions.^16^ The technical details of the PM_2.5_ chamber are presented elsewhere.^23^

A compressor pump (Pump 1), equipped with by a buffer, pressure regulator, dryer, and HEPA filter, provided a stable, dry, and clean air flow to the air filtration test system. The air flow from the compressor pump was delivered to a mass flow controller (Horriba, Stec SEC-500) (Q2) and to the PM_2.5_ chamber (Q1). The Q2 air flow, which was controlled by the mass flow controller, diluted the particle-laden air flow and adjusted the particle concentration in the filter holders. The Q1 air flow, which was adjusted by a needle valve (Valve 1), supplied the air into PM_2.5_ chamber to maintain the incense burning process. A mini pump installed inside the PM_2.5_ chamber transported the incense smoke to the filter holders with the flow of Q4. The Q4 air flow was relatively smaller than that of Q1 so that the excess pressure was released through an opening port with the flow of Q3.

The tested filter was placed inside the filter holder. A differential pressure sensor attached to the filter holder measured the filter pressure drop. A CPC (TSI, 3025a) measured the upstream and downstream particle concentration for calculating the filter efficiency. The upstream particle concentration was measured at the outlet of the dummy filter holder, while the downstream particle concentration was measured at the outlet of the filter holder containing the tested filter.

The CPC had a constant inlet air flow (Q7) of 0.3 L min^−1^ so that the air flow passing through the filter holders (Q6) was adjusted by a compensating air flow (Q8) so that Q6 = Q7 + Q8. A vacuum pump (Pump 2) provided the compensating air flow, which was adjusted by a needle valve (Valve 2) and continuously monitored by a flowmeter (Honeywell, AWM5101VN). To maintain the pressure inside the filter holder at ambient pressure conditions, the excess pressure was released through an opening tube with the air flow of Q5. Also, to prevent the ambient air entering the filter holders, Q6 was set lower than Q5 for all the experiments.

## 3. Results and discussion

### 3.1 Fiber morphology

The morphology of the fibers can be changed by adjusting the solution concentration or using a different solvent. These actions greatly affect the properties of the precursor solution, such as viscosity, surface tension, and conductivity, which leads to different fiber or membrane properties.^21,24,25^Table 1 shows the effect of the solution concentration and the type of solvent on the solution viscosity, conductivity, and surface tension.

**Table 1:** The properties of the ABS precursor solution at different concentrations and in different solvents

Solvent	Viscosity (centipoise)			Surface tension (dyne cm^−1^)			Conductivity (μS cm^−1^)		
	10 wt%	20 wt%	30 wt%	10 wt%	20 wt%	30 wt%	10 wt%	20 wt%	30 wt%
DMAc	13.8	158.1	1672.6	41.2	39.1	37.7	11.5	16.2	19.4
DMF	13.5	185.2	1718.3	42.3	41.4	41.0	11.8	16.3	19.8
THF	12.9	238.8	1840.3	38.6	37.2	35.8	10.6	14.4	18.9

Based on Table 1, for all types of solvents, an increase in solution concentration causes a significant increase in solution viscosity. Conversely, the surface tension decreases as the concentration increases. These results are similar to those of previous studies that correlated the concentration of polymers to the solution viscosity and surface tension.^21^ The conductivity also increased with increasing polymer concentration, which was in accordance with several previous studies.^18,26^ Briefly, the effect of the solution concentration using the ABS waste polymer on the viscosity, conductivity, and surface tension had a similar trend as those using pure polymers.

**Fig. 3:**
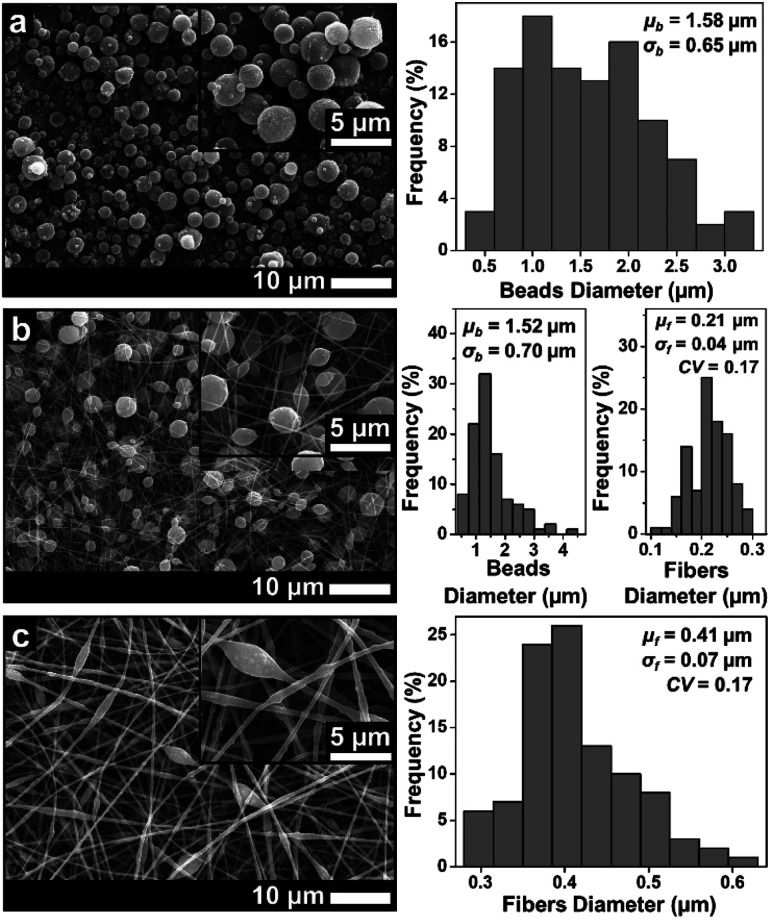
SEM images of membranes using DMAc solvent with concentrations of (a) 10 wt%, (b) 20 wt%, and (c) 30 wt%.

**Fig. 4:**
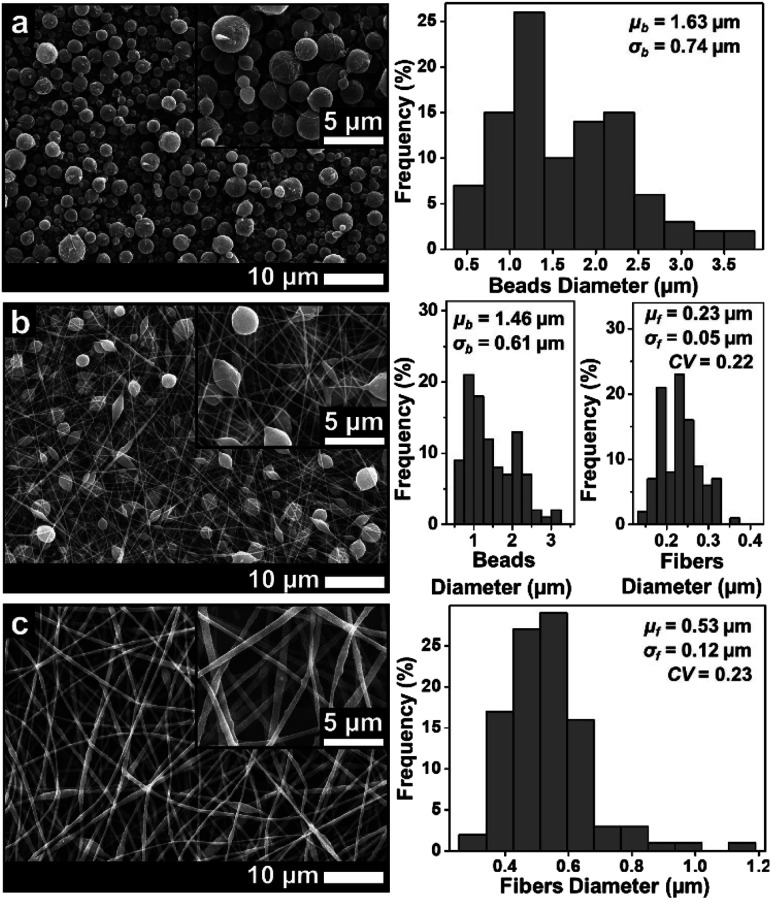
SEM images of membranes using DMF solvent with concentrations of (a) 10 wt%, (b) 20 wt%, and (c) 30 wt%.

**Fig. 5:**
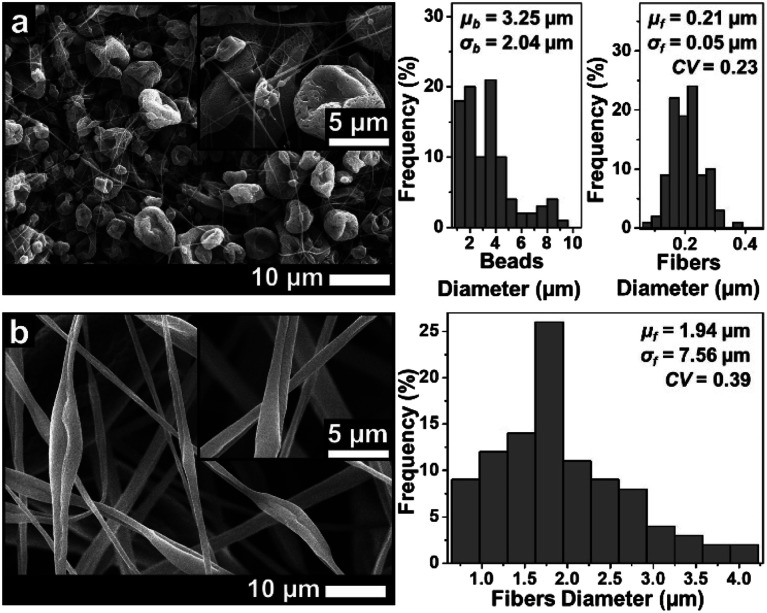
SEM images of membranes using THF solvent with concentrations of (a) 10 wt% and (b) 20 wt%.

**Fig. 6:**
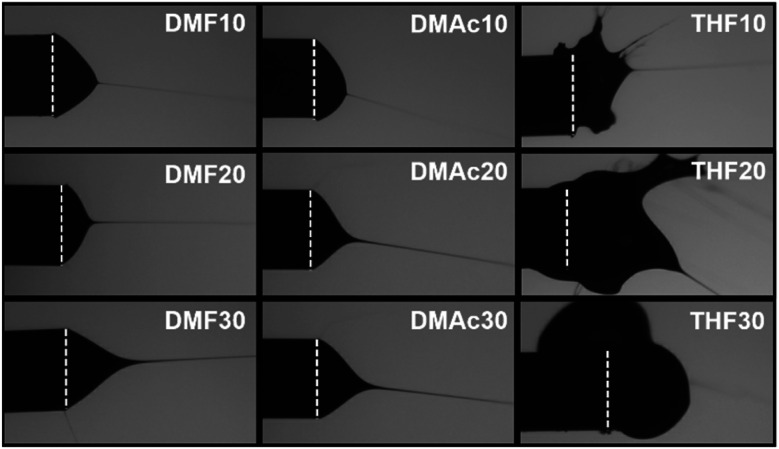
Forms of the cone-jet during the electrospinning process. The dashed lines show the tip of the needle.

Fig. 3–5 show the fiber morphology variation of the fabricated ABS membranes as an effect of the increasing ABS concentrations in DMF, DMAc, and THF solvents. We obtained at least three morphologies, namely pure fiber, beaded fiber, and beads. The shapes of the cone-jet from each solution variation during the electrospinning process are shown in Fig. 6.

Fig. 3 depicts the morphological changes from beads to beaded fibers as the effect of increasing solution concentration for the ABS solution using DMAc solvent. When the solution concentration was 10 wt%, the beads were produced with a *μ*_b_ of 1.58 μm (see Fig. 3(a)). The increase of the solution concentration to 20 wt% changed the morphology to the beaded fiber with a *μ*_f_ of around 0.21 μm (see Fig. 3(b)). Furthermore, when the solution concentration was increased to 30 wt%, a *μ*_f_ increased to 0.41 μm and the number of beads decreased significantly (see Fig. 3(c)). Some researchers also reported that the morphological changes from beads to pure fibers was closely related to the solution concentration.^18,27^ The low solution concentration makes a low viscous solution, which limits the attractive forces among the polymer chains, so they cannot form chain attachments.^12,27^ In this case, the solution surface tension and conductivity dominantly cause the jet breakdown and bead formation.^12,27^

The increase of the solution concentration to 20 wt% caused a significant increase in the solution viscosity, which was about 10 times higher than that for solution of 10 wt% (see Table 1). The high viscosity caused an increase in the attractive force among the polymer chain so that it could overcome the extension by the coulombic force and change the morphology from beads to pure fibers. However, the existence of the beads along the fibers indicated that the surface tension still had a strong enough force to encounter the viscoelastic stresses.^27,28^ Increasing the solution concentration to 30 wt% also gave a significant increase to the viscosity (see Table 1), which led to an increase of the fiber diameter and a decrease of the number of beads.

Similar to the solution with the DMAc solvent, the solution with the DMF solvent produced beads to pure fiber morphologies as shown in Fig. 4. At a concentration of 10 wt%, the beads morphology was formed with a *μ*_b_ of 1.63 μm. Some fine, small-sized fibers were also found among the beads. When the concentration was increased to 20 wt%, the beads had completely disappeared and the beaded fibers were formed with a *μ*_f_ of 0.23 μm. Finally, when the concentration was 30 wt% (Fig. 4(c)), the beaded fibers were formed with a *μ*_f_ of 0.53 μm. Based on Fig. 3 and 4, the solution with the DMAc solvent produced fibers with more beads and smaller fiber diameter than that produced by the DMF solvent. This was because the solution with the DMF solvent had a higher viscosity and a lower surface tension compared to the solution with the DMAc solvent at the same concentration (see Table 1).

Based on Fig. 5(a), the solution using the THF solvent with a concentration of 10 wt% produced beads (*μ*_b_ = 3.25 μm) surrounded by some fine fibers (*μ*_f_ = 0.21 μm). Furthermore, the solution with 20 wt% produced pure fibers morphology with a *μ*_f_ of 1.94 μm. When the concentration was 30 wt%, no fibers were formed on the collector due to the solution blockage on the needle tip (see Fig. 6).

Among the three solvents, the THF solvent produced the solution with the highest viscosity (see Table 1) because the solvent could expand the polymer chains better.^29^ This indicated that the THF was the best solvent to dissolve the ABS waste completely. However, during the electrospinning, the solutions using THF solvent was more difficult to process than the other two solvents because of its lower boiling point (THF = 66 °C, DMF = 153 °C, DMAc = 165 °C). The low boiling point led to a faster evaporation rate. During the electrospinning process, the high evaporation rate caused the polymer solution to experience thermodynamic instability, which led to a separation between the polymer-rich and polymer-poor phases that eventually formed beads with arbitrary shapes (see Fig. 5(a)).^30^ A high evaporation rate also caused a blockage on the tip of the needle (see Fig. 6). At a concentration of 20 wt%, the blockage of the dried solution made an unstable jet formation that caused the variety of fiber sizes (CV = 0.39), as shown in Fig. 5(b). At a concentration of 30 wt%, the high evaporation rate and high viscosity of the precursor solution (see Table 1) caused a total blockage on the tip of the needle so that no fiber was formed on the collector.

### 3.2 FTIR spectra

**Fig. 7:**
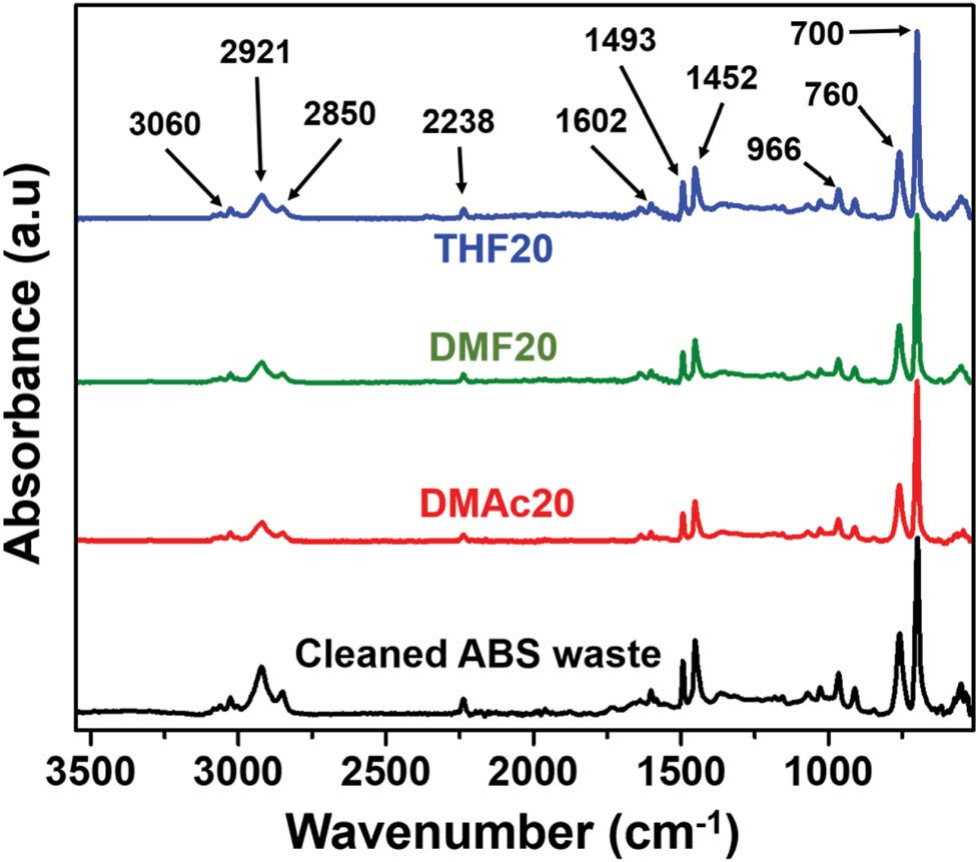
FTIR spectra of the ABS membranes fabricated using various solvents and the cleaned ABS waste.

Fig. 7 shows the absorbance band characteristics of the ABS membrane samples of THF20, DMF20, and DMAc20, as well as the cleaned ABS waste. The absorbance bands characteristics of the ABS are ∼3060 to ∼2850 cm^−1^ (C–H stretching of CH, CH_2_ and aromatic rings), ∼2238 cm^−1^ (C≡N stretching), ∼1602 cm^−1^ (*cis*-1,4-C=C stretching), ∼1450 to ∼1500 cm^−1^ (CH bending of aromatic ring), 966 cm^−1^ (*trans*-1,4-C=C stretching), 760 and 700 cm^−1^ (mono-sub phenyl group).^31^

The DMF solvent has peaks at ∼1673, ∼1389, ∼1256, and ∼1096 cm^−1^.^32^ The presence of DMAc solvent relates to the peaks at ∼1633, ∼1392, ∼1011, and ∼2931 cm^−1^.^33^ The THF solvent has the peaks at ∼3000, ∼1450–1400, and ∼1100–1000 cm^−1^.^34^ We also conducted FTIR measurements to the DMF, DMAc, and THF solvents and those peaks appeared in the FTIR spectra of the solvents (see Fig. S1 in the ESI†). However, none of those peaks appeared on the FTIR spectra of the fabricated nanofiber membranes. The fabricated nanofiber membranes also had similar peaks to those of the cleaned ABS waste. This indicates that all solvents fully evaporated during the electrospinning process.

### 3.3 Membrane wettability

Knowing the membrane surface wettability (hydrophilic or hydrophobic) is important for determining the suitable application of the membrane. Membranes with a hydrophilic surface (CA < 90°) are ideal as water filtration media,^35,36^ while membranes with a hydrophobic surface (CA = 90–150°) are more suitable as air filtration media.^12^ The hydrophobic membranes can also be used as an oil and water separator because membranes with the hydrophobic surface are usually oleophilic.^37^ Besides the hydrophilic and hydrophobic properties, a membrane surface can be categorized into superhydrophilic (CA < 5° in 0.5 s) and superhydrophobic (CA = 150–180°),^38^ though we do not discuss them in this paper.

**Fig. 8:**
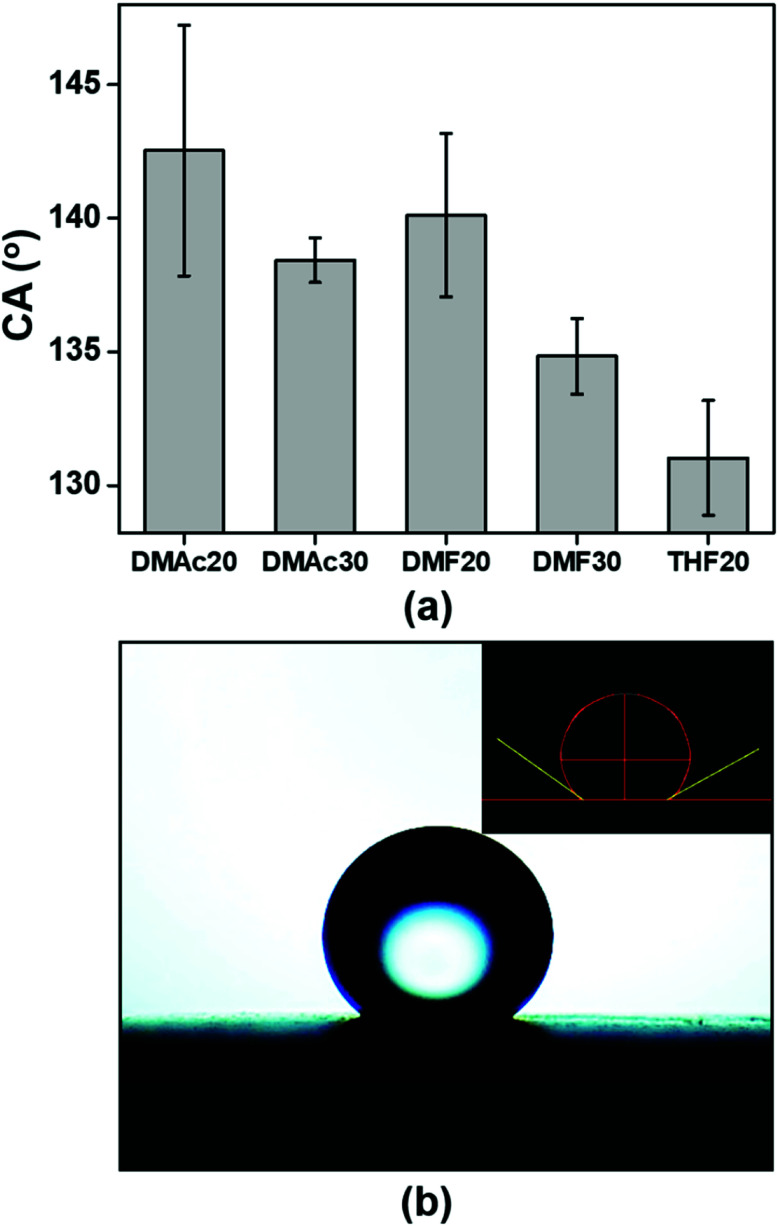
(a) Water contact angles (CA) of the ABS nanofiber membranes and (b) an image of a water droplet on the surface of an ABS nanofiber membrane (DMAc20).

Fig. 8 provides information about the wettability of the ABS membranes (DMAc20, DMAc30, DMF20, DMF30, and THF20) using water contact angle measurements. The figure shows that the DMAc20 membrane has the highest CA of 142.5 ± 4.7, followed by DMF20, DMAc30, DMF30 and THF20 membranes with contact angle values of 140.1 ± 3.1, 138.4 ± 0.8, 134.8 ± 1.4, and 131.0 ± 2.1, respectively. These results indicated that all the fabricated membranes have a hydrophobic property (CA > 90°).^39^ The slight difference in the CA value of each membrane was more due to the influence of the fiber morphology. According to the Cassie model, the rougher surface may decrease the wettability and increase the water contact angle.^40^ Considering the number of beads or bead density of the beaded fibers samples, the SEM images in Fig. 3–5 show that the DMAc20 and DMF20 membranes have a higher bead density than those of the other membranes. The beads directly affect the surface roughness, such that a higher bead density makes the surface rougher. The presence of the beads also facilitates air being trapped between the surface and the water droplets when the water droplets are dropped on the surface, leading to a higher contact angle.^12,21,40^ Therefore, controlling the bead density can directly control the surface roughness as well as the wettability of the membranes.

### 3.4 XRD

**Fig. 9:**
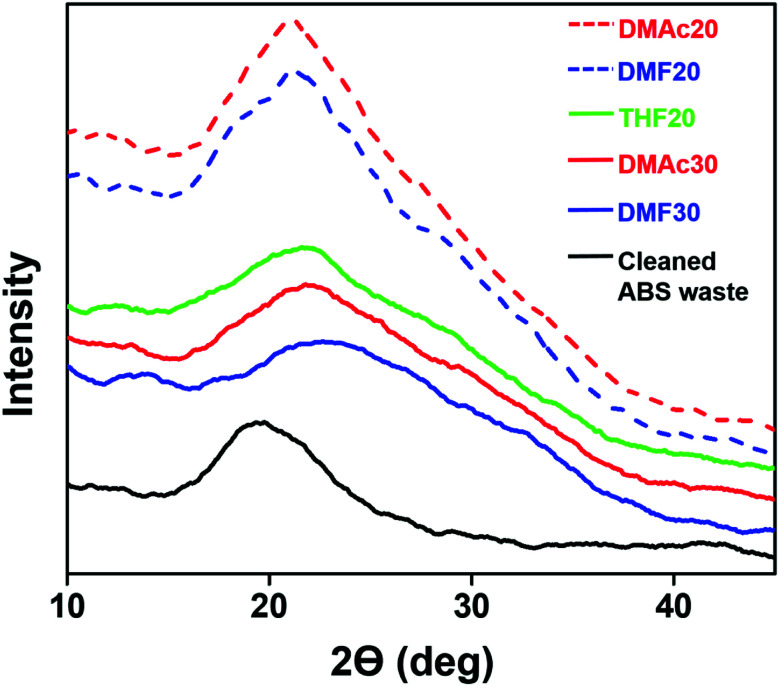
X-ray diffractograms of the fabricated ABS membrane samples and the cleaned ABS waste.

Fig. 9 shows the XRD patterns of the fabricated ABS membranes (DMAc20, DMAc30, DMF20, DMF30, and THF20) and the cleaned ABS waste.

Pure ABS has been reported to have an amorphous structure for the copolymer with a wide main peak at ∼19.7°.^4^ This was similar to our XRD result from the cleaned ABS waste (see Fig. 9). When the ABS waste was in the nanofiber form, its structure remained amorphous for the three solvents. This was reasonable because when the solution was attracted to the collector due to the coulombic force, the solvent evaporation was done in a short time so that the crystallization process did not proceed properly, which led to the formation of an amorphous structure.^41^ In addition, several studies reported that there is an influence of solvent evaporation rate on the fiber diameter, molecular orientation, and crystallinity. A slower evaporation rate causes a longer fiber relaxation or elongation process, which makes the fibers have a smaller diameter, higher molecular orientation degree, and better crystallinity.^42,43^ Accordingly, the THF20, DMAc30, and DMF30 samples had wider peaks than those of the other membranes. The wide peak of THF20 was because of the high evaporation rate, while the wide peak of DMAc30 and DMF30 were likely caused by their high solution concentration.

### 3.5 Tensile strength

**Fig. 10:**
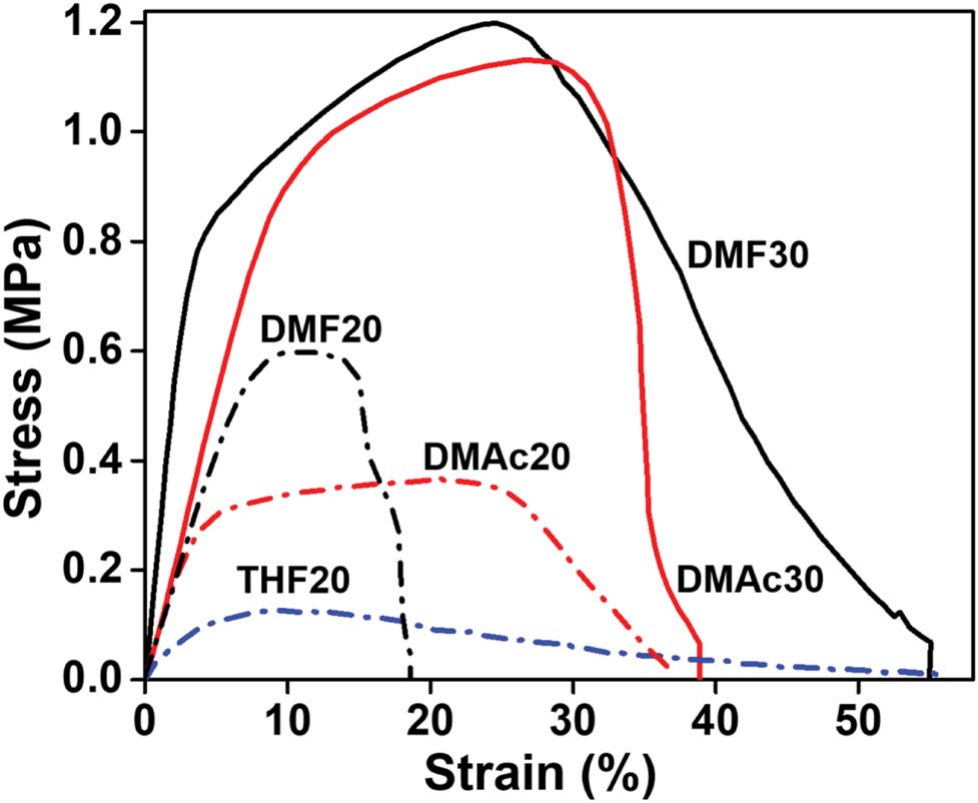
Stress–strain curves of the fabricated nanofiber membranes.

The solvents for making the precursor solutions influence the fabricated nanofiber size and morphology, which later determine the membrane mechanical properties.^44^ The stress–strain curve of the fabricated nanofiber membranes is shown in Fig. 10.

Based on Fig. 10, the DMF30 membrane had the best ultimate tensile strength (UTS) of 1.1 ± 0.1 MPa with an elongation at break (*dl*) of 55 ± 3%. The DMAc30 membrane had a slightly smaller UTS of 0.9 ± 0.1 MPa and *dl* of 41 ± 2%. A significant decrease in the UTS and *dl* values occurred for all membranes made from lower solution concentrations, *i.e.* DMF20, DMAc20, and THF20. The UTS of DMF20, DMAc20, and THF20 were 0.6 ± 0.05, 0.5 ± 0.1, and 0.2 ± 0.1 MPa, respectively. While the *dl* values of DMF20, DMAc20, and THF20 were 19 ± 2, 35 ± 3, and 61 ± 12%, respectively.

Many factors can affect the mechanical properties of the electrospun nanofiber membranes, including fiber diameter, fiber morphology, fiber size uniformity, and fiber composition.^45^ Papkov *et al.* have reported a significant increase in the mechanical properties of PAN nanofibers due to a decreasing fiber diameter.^46^ Similarly, Xu *et al.* reported that a smaller fiber diameter had a better molecular orientation and crystallinity, which resulted in a better mechanical strength.^42^ This explained why the THF20 membranes had the worst mechanical strength, considering it had the largest fiber diameter of 1.94 μm.

Contrarily, the DMF30 membranes (*μ*_f_ of 0.53 μm) had a better mechanical strength than DMAc30 (*μ*_f_ of 0.41 μm), DMF20 (*μ*_f_ of 0.23 μm), and DMF20 (*μ*_f_ of 0.21 μm) membranes, even though DMF30 had a larger fiber diameter. This situation might be associated with the presence of the beads. The beads inside the membranes reduced the interactions among the fibers and led to a low fiber cohesion point that reduced the mechanical performance of the membranes. In this condition, beads could be considered as a defect in the membranes, which reduced the mechanical performance of the membranes.^12,44,45,47^ Based on the SEM images in Fig. 3–5, the membranes made from low solution concentrations tended to have a smaller fiber diameter with a higher bead density that caused low mechanical strengths of DMF20 and DMAc20 membranes compared to those of DMF30 and DMAc30. Similar results have been reported by Tarus *et al.*^44^ Increasing the solution concentration is expected to increase the mechanical strength of the produced membrane until a certain point. After that point, the mechanical strength reduces with an increase of solution concentration due to the production of fibers with a large diameter.

DMF30 had the highest Young's modulus (14.6 ± 1.5 MPa) followed by DMAc30 (9.8 ± 0.9 MPa), DMF20 (8.4 ± 0.4 MPa), DMAc20 (8.0 ± 0.8 MPa), and THF20 (3.4 ± 0.9 MPa). The fabricated membranes with a high tensile strength tended to have a high Young's modulus, which was in accordance with the results reported by Fadaie *et al.*^45^ The lower Young's modulus of the membrane samples fabricated with a lower solution concentration, such as DMF20, DMAc20, and THF20, were obvious because lower concentrations led to smaller fiber diameters. Membranes arranged by small size fibers usually have less stiffness.^44^

### 3.6 Air filtration

#### 3.6.1 Filter samples

Based on the previous results, the membranes produced using DMAc and DMF solvents had a small and homogeneous fiber size, high-water contact angle, and better mechanical properties compared to those of the produced THF solvents at the same concentration. Accordingly, we considered that the membranes fabricated using DMAc and DMF solvents were more suitable as air filter media. Therefore, for the air filtration performance test, we conducted a further experiment specifically for the membranes produced using DMAc and DMF solvents. We tested some ABS membranes fabricated using the DMAc solvent with a solution concentration of 20, 25, 30, and 35 wt% and the DMF solvent with solution concentrations of 20 and 30 wt%.

**Fig. 11:**
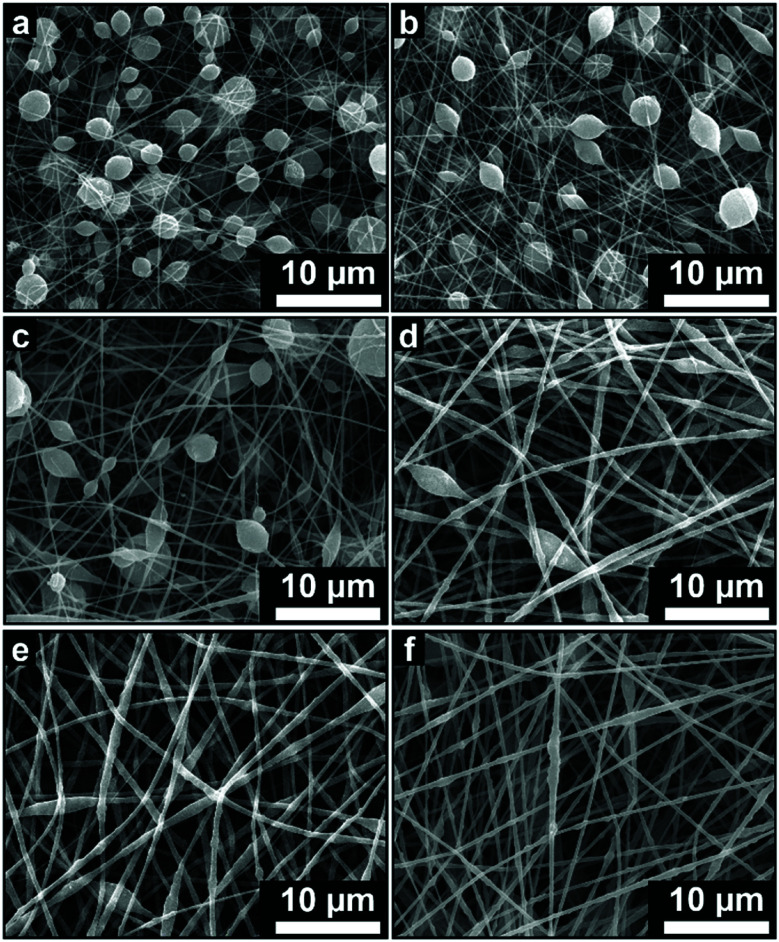
SEM images of ABS nanofiber membranes of (a) DMAc20, (b) DMF20, (c) DMAc25, (d) DMAc30, (e) DMF30 and (f) DMAc35.

Table 2 provides information about the fiber diameter and morphologies of the ABS membrane samples for air filtration performance test. Fig. 11 shows the morphological changes from beaded fiber to pure fiber as an effect of the increasing concentration. Each fabricated membrane sample had a different property that represents four conditions, *i.e.* fiber with a high bead density (Fig. 11(a) and (b)), intermediate bead density (Fig. 11(c) and (d)), low bead density (Fig. 11(e)), and no beads (Fig. 11(f)).

**Table 2:** The properties of the ABS membranes with DMAc and DMF solvents

Sample	Concentration (wt%)	Average fiber diameter (μm)	Fiber morphology
DMAc20	20	0.21 ± 0.04	Beads
DMF20	20	0.23 ± 0.05	Beads
DMAc25	25	0.29 ± 0.05	Beads
DMAc30	30	0.41 ± 0.07	Beads
DMF30	30	0.53 ± 0.12	Beads
DMAc35	35	0.44 ± 0.11	No beads

#### 3.6.2 Filtration performance test

**Fig. 12:**
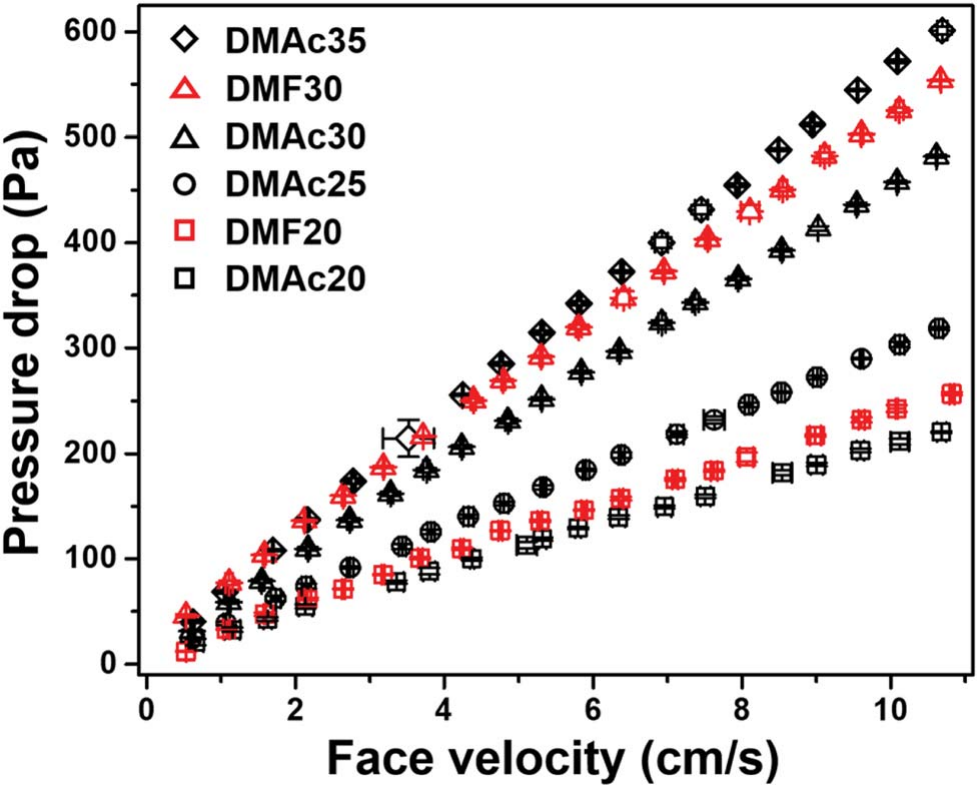
Pressure drop *vs.* face velocity of the DMAc20, DMF20, DMAc25, DMAc30, DMF30, and DMAc35 membranes.

A good air filter should have a high filtration efficiency and low pressure drop. A filter with a high-filtration efficiency tends to have a high pressure drop and *vice versa*. The quality of the filters based on their efficiency and pressure drop is expressed qualitatively by the quality factor value (*q*_F_) as follows:2
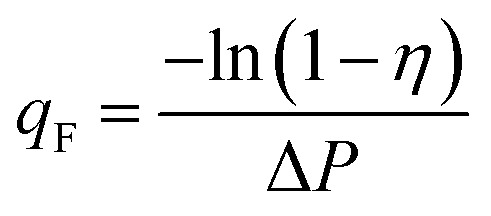
where *η* is the filtration efficiency, and Δ*P* is the pressure drop.^48^Fig. 12 shows the pressure drop as a function of face velocity (=volumetric air flow through the filter/tested filter area) from the DMAc20, DMAc25, DMAc30, and DMAc35 membranes.

Based on Fig. 12, all the membranes show linear dependencies of the pressure drop to the face velocity, which follows Darcy's law.^49^ The membrane with the highest pressure drop was DMAc35, followed by DMF30, DMAc30, DMAc25, DMF20, and DMAc20. The filter pressure drop can be affected by many factors, including fiber diameter, fiber morphology, membrane thickness, and membrane packing density.^22^ Based on Fig. 11, the most striking difference of each membrane sample was their bead density. The presence of beads is responsible for the physical separation among the fibers. A membrane with high a bead density has more space for the air to flow through the membrane, which reduces the membrane pressure drop.^22,50,51^

Table 3 lists the pressure drops, filtration efficiency, and quality factors of the DMAc20, DMF20, DMAc25, DMAc30, DMF30, and DMAc35 membranes. The measurements were carried out using a face velocity of 5.3 cm s^−1^, which is usually used for nanofiber membrane filter test, as by Bao *et al.*,^52^ Matulevicius *et al.*,^22^ and Balgis *et al.*^53^

**Table 3:** The pressure drops, filtration efficiencies, and quality factors of the ABS membranes

Sample	Pressure drop (Pa)	Efficiency (%)	Quality factor (10^−2^ Pa^−1^)
DMAc20	118.7 ± 1.5	95.16 ± 0.08	2.55 ± 0.03
DMF20	135.9 ± 1.2	95.51 ± 0.24	2.28 ± 0.02
DMAc25	168.0 ± 2.0	95.73 ± 0.22	1.88 ± 0.02
DMAc30	251.7 ± 1.5	97.36 ± 0.13	1.44 ± 0.01
DMF30	291.7 ± 1.0	98.19 ± 0.49	1.38 ± 0.01
DMAc35	314.6 ± 2.1	98.39 ± 0.07	1.31 ± 0.01

Based on Table 3, the DMAc35 membrane has the highest efficiency and pressure drop. The absence of beads caused the membrane to have a smaller pore size and higher packing density, which facilitated better particle capture.^50^

Interestingly, the highest *q*_F_ was obtained from the DMAc20 membrane, which had the highest bead density. Although the beads reduced the mechanical performance of the membranes, their presence gave advantages for the filtration performance of the membranes. The beads optimized the structure of the membranes by providing a pathway for the air flow to pass through it easily. It significantly decreased the membrane pressure drop without altering the filtration efficiency too much.

Bao *et al.* reported that the presence of beads could also increase the membrane lifetime. This was because the increased distance among the fibers due to the beads allowed the particle capture process to occur inside the membrane (depth/cake filtration) instead of on the membrane surface.^52^ We conducted experiments to compare the lifetime of the membranes with beaded and pure fiber morphology using a setup similar to that used by Bao *et al.* as shown in Fig. S2 in the ESI.† We delivered incense smoke with a relatively high concentration to the filters while the filter pressure drop was recorded over time.

In contrast to the result obtained by Bao *et al.*, we found that the membrane with a beaded fiber morphology clogged faster than that of the pure fiber morphology, as indicated by the faster rise of the pressure drop (see Fig. S3 in the ESI†). Bao *et al.* observed the depth filtration mechanism using NaCl particles. In our case, we expected that the depth filtration mechanism, as reported by Bao *et al.*, was ineffective at carrying out incense smoke filtration. The incense smoke contains many volatile organic compounds (VOCs), such as benzene, toluene, xylenes, aldehydes, and polycyclic aromatic hydrocarbons.^16^ As reported by Rajak *et al.*, the compounds in the incense smoke led to the formation of a coating layer on the surface of the membranes during the smoke filtration.^13^

From the experimental results, we are optimistic that the filter membranes made from the recycling of ABS waste are good as air filter media for daily needs. For practical applications, the mechanical property of the membranes can be improved by designing a triple-layer structure in which the ABS membrane is placed between two strong nonwoven membranes.^48^

## 4. Conclusions

Nanofiber membranes from ABS waste have been successfully made using the electrospinning method for use as air filtration media. We made the membranes using various solvents (DMAc, DMF, and THF) and solution concentrations. The solvent and solution concentrations affected the fiber size and morphology, wettability, crystallinity, and mechanical properties of the membranes. An increasing solution concentration caused morphological changes from beads to pure fibers. Among the three solvents, the THF solvent is less recommended due to the difficulty of the solution to be processed by electrospinning. The FTIR spectra showed that all the solvents fully evaporated during the electrospinning process. The testing of the membrane surface properties showed that all the fabricated membranes were hydrophobic and the membrane wettability could be adjusted by controlling the bead density. The XRD analysis depicted the amorphous structure of the fabricated ABS membranes. The solvent evaporation rate affected the crystallinity and the arrangement of the molecules, as a higher evaporation rate causes poorer crystallinity and molecular arrangement. From the tensile strength test, the solution using DMF and DMAc with a concentration of 30 wt% has high mechanical strength. The presence of beads could reduce the mechanical properties of the membranes. Overall, the membranes produced using DMAc and DMF solvents met the most suitable criteria for use as air filter media considering the fiber size, wettability, and mechanical properties. We conducted a filtration performance test on some membranes fabricated using DMAc and DMF solvents. The test results depicted that the existence of beads increased the distance among fibers, so that there was more space for air to flow through the membrane, causing a low pressure drop without changing the efficiency too much. These results open the opportunity for utilizing ABS waste on a commercial scale as masks or filters for daily needs.

## Conflicts of interest

There are no conflicts to declare.

## Supplementary Material

RA-009-C9RA04877D-s001
