# Supplementary material for: The synthesis of nanofiber membranes from acrylonitrile butadiene styrene (ABS) waste using electrospinning for use as air filtration media
Source: RSC Adv. 2019 Sep 30;9(53):30741–51. doi: 10.1039/c9ra04877d (PMC9072414; doi:10.1039/c9ra04877d)
Supplement: RA-009-C9RA04877D-s001 [file RA-009-C9RA04877D-s001.pdf]

Supplementary Information

Synthesis of Nanofiber Membrane from Acrylonitrile Butadiene Styrene (ABS) Waste Using Electrospinning as Air Filtration Media

Akmal Zulfi,[a] Dian Ahmad Hapidin,[a] Muhammad Miftahul Munir,[a,b,*] Ferry Iskandar,[a,c] and Khairurrijal Khairurrijal[a,b,**]

[a]Department of Physics, Faculty of Mathematics and Natural Sciences,
[b]Research Center for Biosciences and Biotechnology, Institute for Research and Community Services,
[c]Research Center for Nanosciences and Nanotechnology, Institute for Research and Community Services,
Institut Teknologi Bandung, Jalan Ganesa 10, Bandung 40132, Indonesia

Author for correspondence:
*miftah@fi.itb.ac.id,
**krijal@fi.itb.ac.id

Supplementary Information Results and Discussion

1. The FTIR spectra of the solvents (DMF, DMAc, and THF)

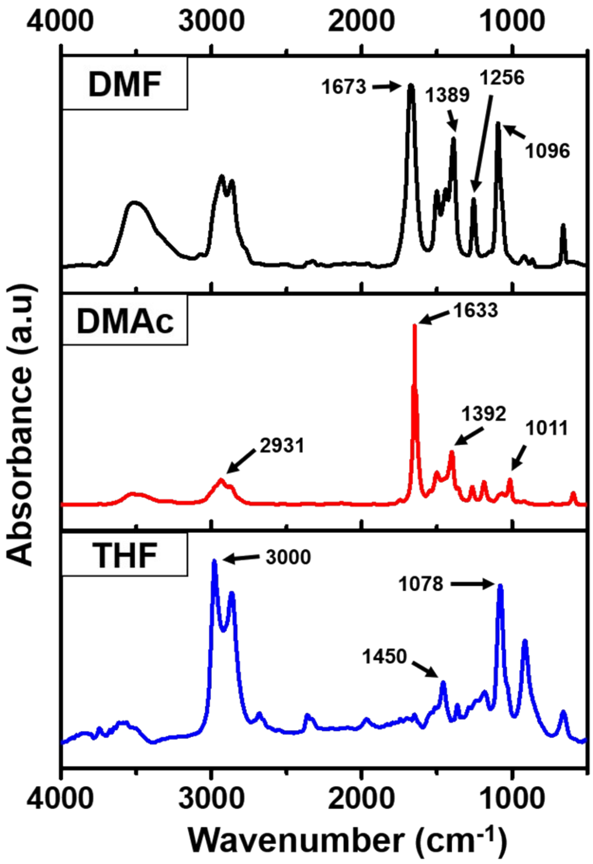

Fig. S1 The FTIR spectra of the DMF, DMAc, and THF solvents.

Please do not adjust margins

2. The experiment for comparing the filter lifetime

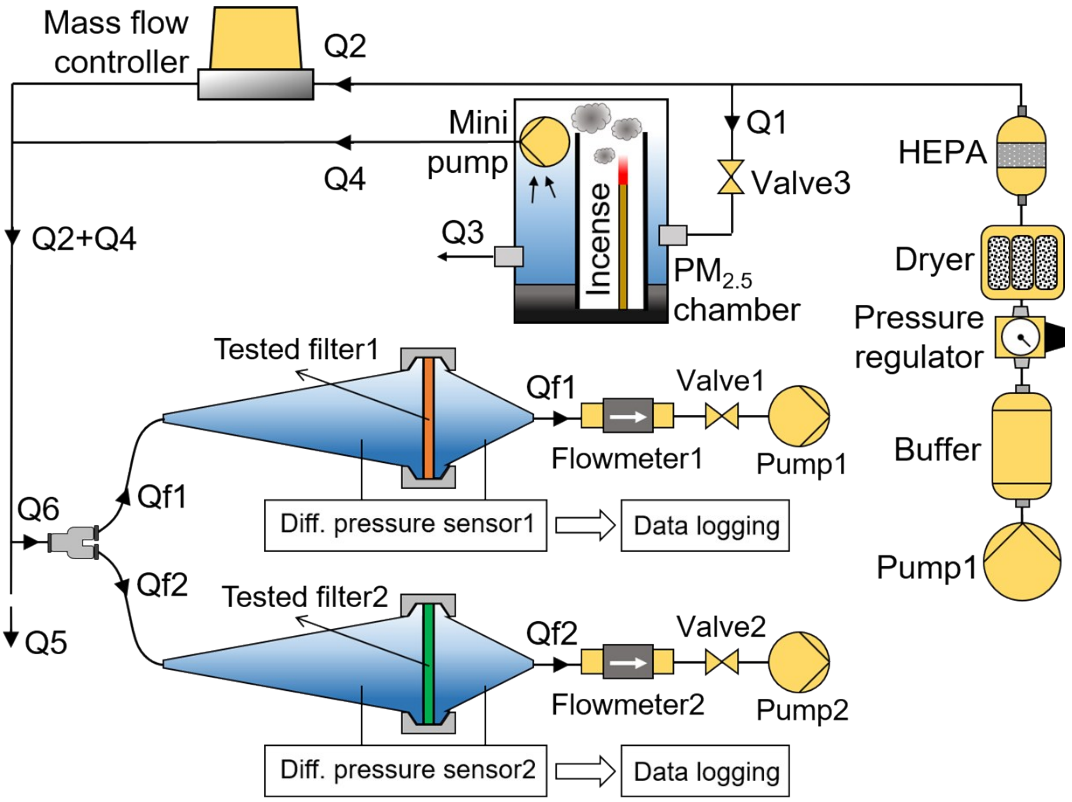

Fig. S2 The experimental setup for comparing the pressure drop of the filter with beaded fiber (Tested filter1) and pure fiber (Tested filter2).

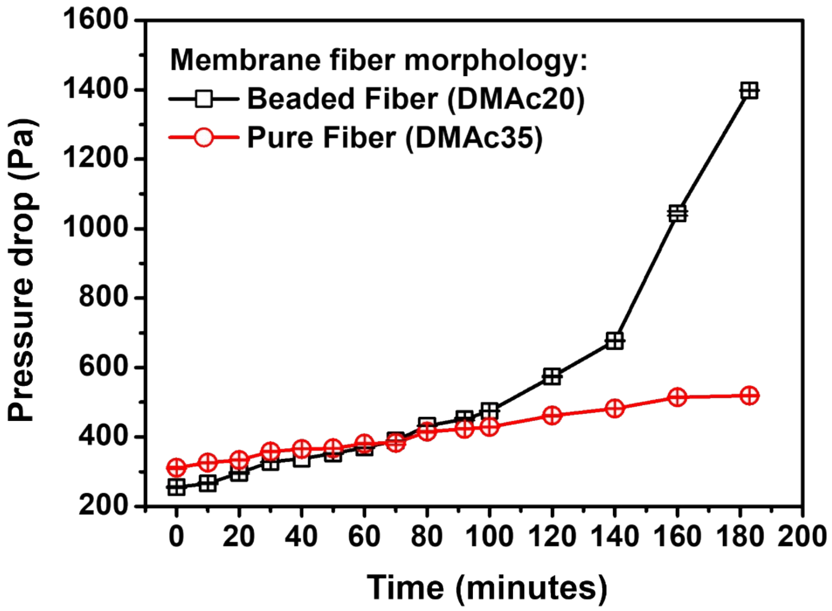

Fig. S3 The comparison of the pressure drop over time for the membrane samples with beaded fiber (DMAc20) and pure fiber (DMAc35) morphologies. The experiment was done using the setup in Fig. S2 with incense smoke as the particle source for the test.

This journal is © The Royal Society of Chemistry 20xx

Please do not adjust margins
